# Interventions for increasing return to sport rates after an anterior cruciate ligament reconstruction surgery: A systematic review

**DOI:** 10.3389/fpsyg.2022.939209

**Published:** 2022-08-22

**Authors:** Kristina Drole, Armin H. Paravlic

**Affiliations:** ^1^Faculty of Sport, Institute of Kinesiology, University of Ljubljana, Ljubljana, Slovenia; ^2^Science and Research Centre Koper, Institute for Kinesiology Research, Koper, Slovenia; ^3^Faculty of Sports Studies, Masaryk University, Brno, Czechia

**Keywords:** return to preinjury, anterior cruciate ligament injury, exercise intervention, psychosocial intervention, injury rehabilitation, return to play

## Abstract

**Background:**

An injury followed by surgery poses many challenges to an athlete, one of which is rehabilitation, with the goal of returning to sport. While total restoration of physical abilities is a primary goal for most athletes, psychosocial factors also play an important role in the success of an athlete's return to sport (RTS). The purpose of this review was to examine the effectiveness of exercise and psychosocial interventions on RTS rates, which might be one of the most important outcomes for elite athletes.

**Methods:**

To carry out this review, PubMed, SAGE Journals, Web of Science, SPORTDiscus, ScienceDirect, and Google Scholar databases were searched from inception to July 2022. The inclusion criteria consisted exercise or psychosocial intervention for athletes after anterior cruciate ligament reconstruction (ACLR), with reporting RTS rates as an outcome.

**Results:**

From 1032 identified articles, four reports (*N* = 130) met inclusion criteria, all of which examined the recovery after ACLR. The mean MINORS score for the included studies was 16.3 ± 6.1, of which non-comparative studies scored 11.0 ± 1.4, while comparative studies scored 21.5 ± 0.7. There were consistent findings for benefits of exercise and psychosocial interventions on RTS rates. Return to preinjury rates in the reviewed studies vary between 63 and 95% with lower % observed in female athletes and with shorter follow-up. Interventional studies reporting RTS rates with a larger sample size and longer follow-up are needed.

**Conclusion:**

Physical and psychological function, as well as social support can be influenced by appropriate interventions, indicating future work on rehabilitation programs for return to preinjury might consider taking the holistic approach addressing those.

## Introduction

While sport and exercise are known to induce physical and psychological health benefits (Ruegsegger and Booth, 2018), sports injuries are an inevitable consequence of participation in elite and recreational sport. Although the risk of sustaining certain injuries can be reduced by ≥50% (Aaltonen et al., 2007) with various injury prevention strategies (Hughes et al., 2018), there is still a number of injuries that cannot be prevented. Athletes who suffer a traumatic injury, particularly one that requires surgery, typically participate in a thorough rehabilitation programme with a view to return to their sport. However, despite often making a full physical recovery, many of these athletes do not return to any level of sports participation, let alone reach their preinjury level (Ardern et al., 2014b).

A range of terms are used throughout the literature to describe the process of an athlete recovering from an injury and returning to full competition. The most common terms used are “return to sport” (RTS) and “return to play” (RTP), which are usually considered as a criteria-based progression from “return to participation” to “return to sport” to “return to performance at preinjury level”. In a recent consensus statement, Meredith et al. (2020) defined “return to participation” as an athlete's return to the training process, but at a lower level than preinjury. In this stage, the athlete is physically participating in training, but has residual psychological, medical, or physical deficits that make him not yet ready to RTS (Meredith et al., 2020). “RTS” refers to an athlete's return to the prior sport, while his performance is not yet at the preinjury level, and “return to performance” describes an athlete returning to their preinjury performance level (Meredith et al., 2020). Throughout this review, the term “return to preinjury level” refers to this final stage of progression. These terms are often used when the athlete is recovering from an injury and can describe successful RTS process. Although, the precise definition can differ from one athlete to another (Meredith et al., 2020).

Moreover, some injuries are classified as more “serious”, “traumatic”, or even “time-loss” as they require a longer rehabilitation, resulting in longer absence from sport, which affects RTS rates as well. A common sports injury of this type is an anterior cruciate ligament (ACL) injury (Montalvo et al., 2019). ACL injuries in elite athletes can affect their ability to continue competing and have other long-term physical and psychosocial consequences, such as development of knee osteoarthritis, inability to earn money and fulfill contracts, academic responsibilities and sponsorship opportunities (Buerba et al., 2021). Prior to anterior cruciate ligament reconstruction (ACLR) surgery, 91% of both female and male patients (31.2 years of age on average) feel they will be able to return to their preinjury level of sport (Feucht et al., 2016). However, research shows that 1 year following surgery 66% of athletes (25.8 years of age on average) of all levels (Ardern et al., 2011b) compared to 83% (Lai et al., 2018) of elite athletes, return to modified training program and limited competition (Ardern et al., 2011b) and only 55% eventually return to their preinjury competition level (Ardern et al., 2014b). However, in most of these studies, the male sex was predominated, suggesting more research is needed in female cohorts to gain the insights in their RTS rates. Furthermore, athletes who return to their preinjury levels have a greater chance of reinjuring their ACL than those who do not return (Webster et al., 2014; Grindem et al., 2016). Along with the physical changes associated with the transition between the state of injury and health, athletes also experience psychosocial changes (Slimani et al., 2018). Common feelings experienced by these athletes include fear of reinjury and losses of identity, support, or motivation, all of which might persist through RTS or even cause the termination of their sports career (Nyland et al., 2002; Kvist et al., 2005; Ristolainen et al., 2012). Research shows that in about 5% of female and male athletes (Ristolainen et al., 2012) to 20% (Kettunen et al., 2001) of elite male athletes, injury is the main reason for career termination, although some studies also report a higher percentage, such as 40 (Drole, 2020) and 50% (Drawer and Fuller, 2001).

Although standard rehabilitation regimens proved to be effective in improving range of motion (ROM) (Shelbourne et al., 2012a,b), recovering muscular strength (Schmitt et al., 2012; Pietrosimone et al., 2013), activation, postural stability, and movement biomechanics, they are usually less effective for returning athletes to preinjury levels of performance (Bien and Dubuque, 2015; Christino et al., 2015). Furthermore, the injury rehabilitation for elite athletes still mostly relies on guidelines (Myklebust and Bahr, 2005), recommendations (Taberner et al., 2019), consensus (Meredith et al., 2020), and clinical commentaries (Bien and Dubuque, 2015) that are considered low level of scientific evidence. Traditionally, a restoration of physical abilities has been a primary focus for athletes and practitioners following the ACL injury and subsequent RTS decision making (Kvist, 2004). However, more recent research also shows that psychosocial factors play an important role in whether or not athletes are able to RTS after an injury (Ardern et al., 2013; Christino et al., 2015; Kunnen et al., 2019, 2021). Hence, these psychosocial factors could partly explain why the athletes are not returning to play despite being physically rehabilitated considering current RTS guidelines. Recent research demonstrated that higher fear of reinjury is negatively associated with stiffened lower limb movement patterns during jumping task (Trigsted et al., 2018), and self-reported physical function (Chmielewski et al., 2008) after ACLR. Later studies support further findings showing that athletes which do not return to their preinjury level of performance have greater fear of reinjury (Kvist et al., 2005) and loss of motivation (Podlog and Eklund, 2005), which can negatively influence the effectiveness of physical therapy rehabilitation and recovery (Podlog and Eklund, 2005; Hsu et al., 2017). The aforementioned factors can be addressed with the use of different techniques (Kunnen et al., 2021), such as motor imagery (Johnson, 2000; Cupal and Brewer, 2001; Lebon et al., 2012; Maddison et al., 2012), goal setting (Evans and Hardy, 2002), modeling videos (Maddison et al., 2006), or virtual reality (Gokeler et al., 2016). It was found that relaxation and guided imagery led to significant improvements in mood scores (Johnson, 2000), lower pain, reinjury anxiety accompanied with greater knee strength in non-elite athletes (Cupal and Brewer, 2001). Further research showed significant differences in knee laxity score and decrease in the level of noradrenaline and dopamine, indicating lower levels of stress in the group receiving imagery intervention (Maddison et al., 2012). Nonetheless, motor imagery was also found to be effective in facilitating greater muscle activation when compared to standard physical therapy alone (Lebon et al., 2012; Paravlic et al., 2020a). The goal-setting intervention resulted in higher self-reported adherence to the rehabilitation program and higher levels of self-efficacy (Evans and Hardy, 2002), while Johnson (2000) did not find any benefits of this kind of intervention in both male and female non-elite athletes. Cognitive based interventions such as motor imagery and action-observation have been found beneficial for improving patients' physical function following major lower limb surgeries (Maddison et al., 2006; Paravlic et al., 2020b; Paravlic, 2022). For example, only two pre-operative action observation sessions resulted in reduced perceptions of expected pain preoperatively and improved self-efficacy during rehabilitation exercises immediately following ACLR surgery (Maddison et al., 2006). Furthermore, the ACLR patients in the experimental group needed crutches for significantly less time (5.54 days) compared to control group (CG) (9.34 days), while they also achieved better scores on International Knee Documentation Committee assessments, showing less functional disability at 6 weeks compared to the CG (Maddison et al., 2006).

Despite high RTS rates following ACLR in athletes, studies show that only few athletes are able to return to preinjury level of performance, which implies a need for interventions to increase return to preinjury rates (Bien and Dubuque, 2015; Christino et al., 2015). Although above mentioned studies show promising results, their effectiveness on RTS rates needs to be further examined. Only a few experimental studies (Arundale et al., 2018; Capin et al., 2019; Coronado et al., 2020; Joreitz et al., 2020) investigated the effectiveness of an intervention on RTS rates. Those have high levels of methodological heterogeneity, therefore, the existing literature needs to be evaluated by using a systematic review approach. Therefore, the main aim of this review was to examine the effectiveness of both physical and psychosocial interventions for RTS rates after ACLR surgery in athletes.

## Materials and methods

The review was conducted in accordance with the PRISMA 2020 guidelines (Page et al., 2021).

### Search strategy

An initial systematic literature search was conducted by one author (KD) in January 2022. Updated search was conducted on 8th July 2022 by two authors (KD and AP) to include new relevant studies. Both the initial and updated searches included following databases: PubMed, SAGE Journals, Web of Science, SPORTDiscus, ScienceDirect, and Google Scholar. No restriction on the year of publication or language were used. The review was not registered in the register of systematic reviews. Upon the first search, it was discovered that the most frequently investigated injury was ACL injury. As a result, the terms “anterior cruciate ligament injury,” and “anterior cruciate ligament reconstruction,” were added to the broader terms of “sport injury” and “sports injury”. The search terms included in the PubMed search strategy were: “exercise intervention”, “psychosocial intervention”, “intervention”, “exercise program”, “program”, “sports injury”, “musculoskeletal injury”, “anterior cruciate ligament reconstruction”, “return to sport”, “return to play”, “RTS”, “RTP”, “return to preinjury”, and their variations, while other databases were searched using the same keywords. The first 100 results from a Google Scholar literature search were screened, as per previously established protocol (Haddaway et al., 2015).

### Eligibility criteria for selecting studies

In accordance with the PICO (population/problem, intervention, comparison, outcome) approach format (Page et al., 2021), inclusion criteria consisted of: (Population) athletes sustaining ACL who were operatively managed with ACLR, (Intervention) an exercise or psychosocial interventions was studied, (Outcome) RTS rates.

Studies were excluded according to the following criteria: (1) studies that did not examine post-sports injury exercise or psychosocial interventions, (2) research on concussion and other non-musculoskeletal injuries, (3) studies conducted on non-athletes, (4) studies in a pediatric population, (5) other than interventional studies, or (6) studies not reporting RTS rates.

### Screening strategy

The initial literature search along with study identification, screening, quality assessment and data extraction were all completed by one researcher (KD). Then, all of the data were independently reviewed by the other author (AP). First, any publications outside the scope of this review were excluded after the reviewers had originally evaluated the titles found through computerized searches. Second, specified inclusion and exclusion criteria were used to evaluate the abstracts. Third, the full texts of the remaining papers that met the inclusion criteria were retrieved and included in the ongoing procedure and reviewed by the two reviewers to reach a final decision on inclusion in the systematic review. The reference lists from the manuscripts that were retrieved were also checked for any additional prospective suitable papers.

### Data extraction

The extracted data from the studies (Table 1) included: study design, injury, population (including patient age and sex), exercise/psychosocial intervention (including rehabilitation stage and duration) and main findings.

**Table 1:** Characteristics of included studies.

**References**	**Study design**	**Population**	**Injury**	**Stage**	**Intervention**	**Duration + follow up**	**Study purpose**	**Main findings**
Arundale et al. (2018)	RCT	40 male athletes mean age = 23.3	ACL	Late: 3–9 months post-op	SAP or SAP+PERT groups of the Anterior Cruciate Ligament-Specialized Post-Operative Return to Sports trial (ACL-SPORTS)	5 weeks, 2x/week + 2-year follow up	Report the RTS and second ACL injury incidence outcomes of the men in the ACL-SPORTS trial.	1 year preinjury: 78% 1-year RTS: 95% 2 years preinjury: 95% 2 years RTS: 100%
Capin et al. (2019)	RCT	39 female athletes mean age = 17.2 ± 2.6	ACL	Late: 3–9 months post-op	ACL-SPORTS	5 weeks, 2x/week + 2-year follow-up	Examine the effect of ACL-SPORTS on strength, hops, function, activity levels, and RTS rates in young female athletes 1 and 2 years after ACLR	2 years RTS: 100% 2 years preinjury: 87%
Joreitz et al. (2020)	Case series	43 (21 completers) male and female athletes mean age = 25.7 ± 8.3	ACL	Early-late	5 phases of criterion-based rehabilitation. Later stages were individualized per requirements of participants' primary sport.	All stages + 2-year follow up	Evaluate RTS and reinjury rates following the criterion-based rehabilitation protocol with a final RTS test that utilizes minimal equipment following ACLR.	2 years RTS: 100% 2 years preinjury: 84%
Coronado et al. (2020)	Pilot study	8 athletes (6 females) mean age = 20.1 ± 2.6	ACL	Early: 8 weeks post-op	7-session telephone-based CBPT-ACLR intervention	8 weeks (7 sessions) + 6-month follow-up	To describe feasibility, adherence, acceptability and outcomes of a CBPT-ACLR intervention for improving postoperative recovery after ACLR.	6-month RTS: 63%

*RCT, randomized controlled trial; CBPT-ACLR, cognitive-behavioral based physical therapy; 1-year RTS, RTS rates at 1 year after ACLR; 1-year preinjury, return to preinjuty level of performance at 1 year after ACLR*.

### Methodological quality assessment

The quality assessment was conducted by one author (KD), while other (AP) checked all data independently. Disagreements were resolved by consensus. For observational or non-randomized studies, the 12-item Methodological Index for Non-randomized Studies (MINORS) was used (Slim et al., 2003). MINORS is a valid instrument and designed to assess the methodological quality of non-randomized studies, whether comparative or non-comparative. Each item was scored a “0” (not reported), “1” (not adequately reported), or “2” (adequately reported). The maximum score was 16 and 24 for non-comparative and comparative studies, respectively.

## Results

**Figure 1:**
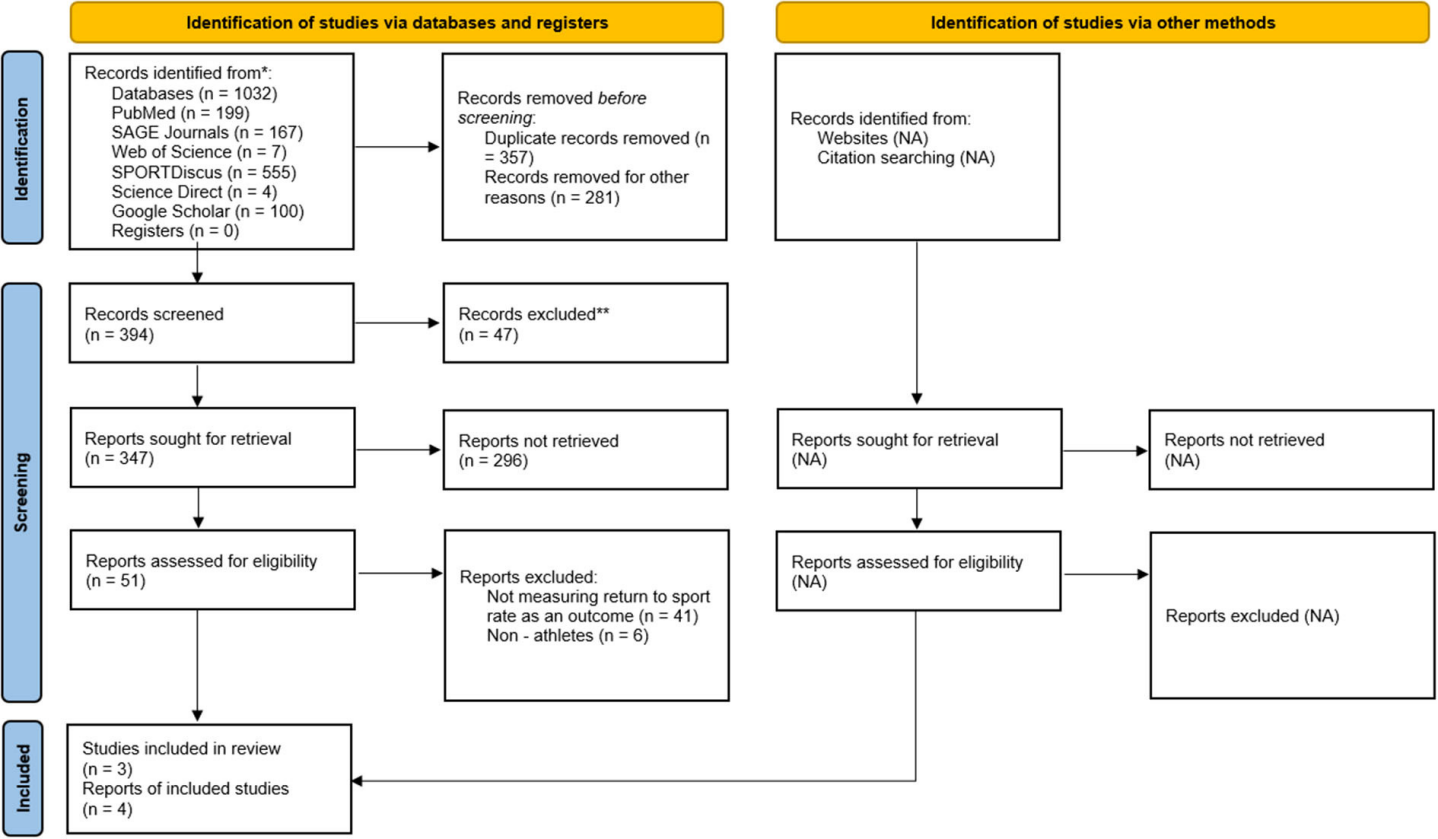
Flow diagram of study selection process.

The initial literature search was conducted in the PubMed, SAGE Journals, Web of Science, SPORTDiscus, ScienceDirect, and Google Scholar databases. Titles were screened from the initial 1032 records acquired through database searches and 47 records were removed based on the exclusion criteria. After abstract screening, an additional 296 reports were eliminated, leaving a total of 51 reports for full text review. Afterwards, 47 articles were removed from the evaluation since they did not assess RTS rates as an outcome or were conducted on non-athletes, leaving four eligible reports for inclusion in the review (Figure 1).

### Quality assessment

The mean MINORS score for the four included longitudinal interventional studies was 16.3 ± 6.1 (Table 2), of which non-comparative studies scored 11.0 ± 1.4, while comparative studies scored 21.5 ± 0.7. All the investigated studies received a maximum of two points for the following items: a clearly stated aim, the inclusion of consecutive patients, the prospective collection of data and adequate statistical analyses of data.

**Table 2:** Methodological quality assessment (MINORS).

**References**	**C1**	**C2**	**C3**	**C4**	**C5**	**C6**	**C7**	**C8**	**C9**	**C10**	**C11**	**C12**	**Total score**	**Max score**
Arundale et al. (2018)	2	2	2	2	1	2	2	0	2	2	2	2	21	24
Capin et al. (2019)	2	2	2	1	1	2	2	2	2	2	2	2	22	24
Joreitz et al. (2020)	2	2	2	2	0	2	0	0	0	0	0	2	12	16
Coronado et al. (2020)	2	2	2	1	0	1	0	0	0	0	0	2	10	16

*C, criterion. C1 A clearly stated aim, C2 Inclusion of consecutive patients, C3 Prospective collection of data, C4 Endpoints appropriate to the aim of the study, C5 Unbiased assessment of the study endpoint, C6 Follow-up period appropriate to the aim of the study, C7 Loss to follow-up less than 5%, C8 Prospective calculation of the study size, C9 An adequate control group, C10 Contemporary groups, C11 Baseline equivalence of groups, C12 Adequate statistical analyses*.

### Types of interventions

All four reports examined recovery after ACLR. Exercise interventions examined secondary injury prevention programs (Arundale et al., 2018; Capin et al., 2019) and criterion-based rehabilitation (Joreitz et al., 2020), including neuromuscular training, strength training, agility drills, and plyometrics, while the psychosocial intervention examined cognitive-behavioral therapy (Coronado et al., 2020). The exercise interventions studies were applied in the early to late rehabilitation stages (Arundale et al., 2018; Capin et al., 2019; Joreitz et al., 2020) while the psychosocial intervention was applied in the early stages of post-operative rehabilitation (Coronado et al., 2020).

### Exercise interventions

Two studies evaluated the effectiveness of ACL-SPORTS program (Arundale et al., 2018; Capin et al., 2019), which consisted of two groups: a strengthening, agility, and secondary prevention group (SAP), and SAP plus perturbation group (SAP+PERT). All athletes had to complete outpatient rehabilitation, be between 3 and 9 months post-surgery, and meet the following criteria for enrolment: 80% quadriceps femoris muscle strength symmetry, minimal knee joint effusion, full ROM, no pain, and able to complete a running progression. The exercises that were chosen upon risk factors for initial ACL injury (Myer et al., 2004, 2006) and subsequent reinjury (Di Stasi et al., 2013) addressed balance and muscle strength impairments and dynamic sport-specific tasks. Plyometric and balance exercises included triple single-legged hops, tuck jumps and box drops, Nordic hamstring exercise and squats with hip abduction were completed in terms of strengthening exercises, while the agility drills were performed as per the University of Delaware guidelines (White et al., 2013). Additional neuromuscular training that included progressive perturbations exercises on unstable surfaces in both bilateral and unilateral stance, was carried out by the SAP+PERT group (White et al., 2013). Both groups underwent 10 training sessions, two times per week for 5 weeks. Arundale et al. (2018) evaluated RTS rates of the men (*n* = 40) from the ACL-SPORTS program. Athletes were found to pass RTS criteria 232 ± 99 days after ACLR. One year after ACLR, 95% of athletes had returned to sport and 78% at their preinjury level. Two years after ACLR all athletes had returned to sport, 95% at their preinjury level and only one athlete had a second ACL injury. At the 2-year follow-up, more athletes in the SAP group had normal knee function when compared to SAP+PERT group according to their IKDC (International Knee Documentation Committee) scores.

Capin et al. (2019) analyzed 39 female athletes from the SAP (*n* = 20) and SAP+PERT (*n* = 19) groups 2 years after primary ACLR. Across these 2 years, nine ACL reinjuries were reported. There was no statistically significant difference in rate or side of second ACL injury between the SAP+PERT and SAP groups. The reinjury rate was 26%, which is not higher than previous research (Webster et al., 2014; Webster and Feller, 2016). Even though the perturbation training performed in the SAP+PERT group did not affect reinjury rates in females, athletes in this study demonstrated amongst the highest RTS rates reported in the literature (100% returned to sport, 87% to preinjury level).

Joreitz et al. (2020) included 43 patients in their study, of which 21 (49%) completed the program and 19 were available for the two-year follow-up. The program consisted of five phases of rehabilitation, starting with becoming independent with activities of daily living, and progressing to running, basic agility training, plyometrics, and rotational cutting and pivoting. In the first phase, the program based on restoring ROM and mobility, relieving pain and edema and implementing the strengthening exercises with a focus on the quadriceps muscles. Phase 2 focused on treadmill jogging, while in the third phase, various agility drills in different body planes were implemented. Fourth phase included jumping with both feet and the fifth phase progressed to one-legged hopping and cutting (Joreitz et al., 2020). Later stages of the rehabilitation program were individualized according to the participants' primary sport requirements. Results showed that at 2 years, 84% of athletes were able to return to their preinjury level of sports competition. A smaller percentage (16%) were able to return to a reduced level of sport and one participant reported a second ACL injury. The average time from surgery to RTS clearance was 10.6 ± 4.4 months.

### Psychosocial interventions

Coronado et al. (2020) introduced a cognitive-behavioral-physical therapy (CBPT) based intervention that was adapted in collaboration between two physical therapists, a clinical psychologist and a sports psychologist with the intent to improve postoperative knee function and the likelihood of RTS. During 8 weeks, eight patients participated in the seven-telephone session intervention with a licensed physical therapist. The following CBPT strategies were utilized: controlled breathing, grounding, setting activity goals, monitoring self-talk, setting daily intentions, present-mindedness, managing setbacks, and guided imagery. At 6 months, five of the eight patients had returned to their primary preinjury sport at varying levels of effort, performance and pain. One patient returned to the same level of sport as before their injury and without pain, while two patients reported pain returning to their same level of preinjury level of effort and performance (Coronado et al., 2020).

### Return to sport criteria

All athletes in the ACL-SPORTS programs (Arundale et al., 2018; Capin et al., 2019) had to pass predetermined return-to-sport requirements to be cleared to return to their preinjury level of performance. These requirements included achieving limb symmetry of 90% in the quadriceps strength test, 90% in each of the four single-legged hop tests, 90% on the Knee Outcomes Survey-Activities of Daily Living scale (KOS-ADLs), and in the general rating of self-perceived knee function (Arundale et al., 2018).

The criteria to RTS in the Joreitz et al. study was defined as follows: all athletes should achieve ≥90% limb symmetry for 1RM strength knee extension, four hop tests, Y-balance test and two functional running tests over a 10-yard course and medical clearance. To return to competition, athletes had to tolerate entire practice sessions including physical contact, performed with maximal effort, without experiencing any pain, swelling, warmth, or episodes of knee giving way (Joreitz et al., 2020).

In the study of Coronado et al., the RTS was assessed by using The SPORTS Score tool. In this questionnaire, three different concepts were evaluated and mainly were categorized as: (1) The ability to perform the same sport at the preinjury level; (2) the ability to perform at the preinjury level of performance; and (3) the ability to perform without the presence of pain (Blonna et al., 2011).

## Discussion

This review aimed to examine the effectiveness of physical and psychosocial interventions on RTS rates after ACLR. We found four interventional studies reporting RTS rates as an outcome. The mean time to passing RTS criteria for male athletes in the ACL-SPORTS program was ~7.5 months after ACLR (Arundale et al., 2018), while for the females in the ACL-SPORTS, average time to RTS was 8.5 months (Capin et al., 2019). Athletes in criterion-based rehabilitation program needed 10.5 months from surgery to RTS (Joreitz et al., 2020). The rates of return to preinjury level in the reviewed studies were 84 and 100%, thus they were higher than those reported in the literature, which typically vary between 40 and 86% (Ardern et al., 2012, 2014a; Brophy et al., 2012; Harris et al., 2013). Higher percentages are usually represented by elite athletes who have greater access to exceptional medical care and attention from support staff compared to non-elite athletes (Lai et al., 2018; Kunnen et al., 2019, 2021). Non-elite athletes report either lack of awareness or lack of finances for professional support during their rehabilitation (Kunnen et al., 2021). Consequently, elite athletes are twice more likely to return to their preinjury level sport, and have six times the odds of returning to competitive sport when compared to non-elite athletes (Ardern et al., 2014b). The results of RTS rates in the ACL-SPORTS cohort are comparable and similar to those of Joreitz et al. (2020) who used criterion-based rehabilitation program. After 2 years, 84% of both female and male athletes managed to return to their preinjury level, which is very similar to the results reported by Capin et al. (2019) who only examined female athletes (87%). However, Joreitz et al. did not exclude participants for any previous knee injury, concomitant knee injuries or procedures at the time of surgical reconstruction, nor for the type of graft or reconstruction procedure they received, while ACL-SPORTS studies (Arundale et al., 2018; Capin et al., 2019) had strict exclusion criteria. This might be the reason for lower RTS rates and longer time passed from surgery to RTS in the criterion-based rehabilitation when compared to ACL-SPORTS studies. It is possible that the rates observed by Joreitz et al. would be different if male and female participants had been analyzed separately. Indeed, after 2 years, fewer female athletes (Capin et al., 2019) returned to preinjury levels compared to males (Arundale et al., 2018) (87 vs. 100%), despite the implementation of identical program. Even though several studies (Podlog and Eklund, 2005; Kunnen et al., 2019, 2021) have investigated psychosocial factors and interventions for RTS following an ACL injury, Coronado et al. (2020) were the only ones reporting RTS rates as an outcome. Despite the small sample size, promising results for enhancing return to preinjury level of sport were observed, as 63% of the athletes had returned to varying levels of sports participation after 6 months.

The inconsistencies observed in the RTS rates could be due to various reasons. One difference would be between males and females, which previous studies have already discussed. Ardern et al. (2014b) found that male athletes were approximately 1.5 times more likely to return to previous level of sport or competitive sport than females, while they found no differences in rate to return to any sport. It was suggested that women after surgery either participate at a lower intensity level, or men are more likely to play competitive sport (Ardern et al., 2014b). It could also be due to differences in motivation for participating in sport (Deaner et al., 2012) or different social roles. For example, a stronger sense of athletic identity may be a positive motivator for returning to sport in those athletes whose lives and social networks mostly revolve around participation in sport (Ardern et al., 2014b). Female sport is still heavily subjected to gender stereotypes, underrepresented in the media and women often receive negative comments from male peers for wanting to participate in sports or for excelling at sports traditionally associated with men, all of which contributes to the lower involvement of women in sport (Deaner et al., 2012). Furthermore, most female athletes cannot ensure their livelihood solely from their athletic careers (Capranica et al., 2013), thus they pursue a dual-career with either being enrolled in the educational process or employed elsewhere besides their sports career. Another concern for female athletes may also be motherhood; when to plan a family and how to combine this with their sports career. Research also shows that female athletes have less financial and social support available (Pfister, 2010), which could result in limited access to sport and medical services that professional male athletes receive. This indicates that the combination of the aforementioned factors could potentially affect the course of rehabilitation and RTS rates. A recent study (Kunnen et al., 2021) also found that fear of re-injury was more common among the older participants, female participants or participants who had experienced more than one injury, which might be the reason for lower RTS rates in these populations. The evidence that also supports these findings is that females try to avoid playing in risky environment-related conditions in order to avoid the chances of getting reinjured (Ardern et al., 2012; Kunnen et al., 2021). Despite the obvious differences in functional, psychological, and social outcomes between females and males following ACLR, both currently receive similar rehabilitative therapies and are assessed using the same clinical criteria to determine readiness for RTS. However, female patients experience more psychological distress, decreased self-efficacy, and increased internal locus of control, while male patients express frustration with their physical limitations but also higher levels of self-efficacy linked to optimism, following ACLR (Lisee et al., 2020; Kunnen et al., 2021). Future interventions should therefore be adjusted according to sex and other factors that influence the course of rehabilitation. Interventions for female athletes should be focused on enhancing their psychological response and self-efficacy, while at the same time reducing fear of reinjury. As well as sex, age is also a confounding factor affecting the outcomes in RTS. Ardern et al. (2014b) found that younger athletes are more likely to return to their preinjury level of sport, which could be due to different priorities, quicker healing processes (Gerstein et al., 1993), while the motivation to continue competing might be higher in youth athletes as well. Sports policy should be focused on trying to bridge the gender gap in sport with making rehabilitation services more accessible to women and youth athletes as well. However, more research is needed to explain the sex and age differences observed in RTS.

While level of competition could also be a reason for differences in RTS rates, the reviewed studies did not report it. It could be that elite athletes are more likely to return to their preinjury level of sport because of their investment in it (longer training hours, financial benefit, scholarships, etc.). At this point, the realization of their goals should also be considered—the athletes who did not yet reach their goals in terms of competition results are more likely to have the motivation required for RTS. Although there are some inconsistencies in the literature, the research suggests that differences in the rate of return to preinjury level sport due to graft types may be small (Ardern et al., 2014b).

Nevertheless, it should be noted that there has been some progress made in RTS rates over the last two decades. Ardern et al. (2011a) found that average RTS rates in studies published before and after 2000 were 78 and 85%, respectively. The difference in rate of return to competitive sport was statistically significant with 44% before 2000 compared with 56% after 2000. While these findings are a promising reflection of recent improvements in applied sports medicine practice, they reveal an important consideration from a research perspective. That is, comparisons of studies conducted several years apart may also be confounded by progressions in equipment, technology and/or general sports medicine understanding, rather than just the nature of the implemented interventions. The studies included in this review were all published within the last 4 years, so it is unlikely that this factor confounds the inferences in this study.

It seems that a secondary injury prevention program with strengthening exercises, agility drills, plyometrics, and sport-specific exercises is effective in addressing the physical impairments after an ACLR. Fear of reinjury appears to be a recurring psychological factor in the studies, which suggests that some form of psychosocial task should be included in future interventions. To date, we don't know about any study that took a holistic approach and included both physical and psychosocial components in the intervention. Future work might consider incorporating exercises that target patient concerns related to fear of reinjury or lack of confidence, possibly in combination with other exercise interventions, to enhance the psychological response. Although the psychosocial interventions are mostly applied in the early stages of post-operative rehabilitation, a major consideration might be the application of these in later rehabilitation stages and after the athletes have RTS. In that period, some limiting factors such as fear and anxiety still persist, thus psychosocial interventions could positively affect these factors, and potentially increase the return to preinjury rate, while reducing the reinjury rate. Successful rehabilitation must be viewed as a complex construct, ensuring safe RTS and lowering the chances of reinjury, rather than just quickly returning the athlete to the competition. Therefore, the practitioners must approach the athlete's rehabilitation holistically, considering his/her physical, psychological and social attributes.

### Limitations and further research

While most studies included male and female athletes of different ages, the reviewed studies only examined athletes after ACLR. Therefore, the results may not be generalizable to athletes recovering from other injuries, indicating a need to investigate other types of traumatic, long-term injuries, followed by a surgery and RTS. Since there are differences in reinjury risk between different sports, things that also need to be considered in RTS are the specifics of the certain sport—such as type (pivoting/non-pivoting, contact/non-contact, and same as preinjury or a different sport), frequency (daily, weekly, monthly, etc.), intensity (recreational, professional), and the performance level (Meredith et al., 2020). More research is needed on sex differences in sport, which impact recovery and RTS after major injuries and the resources available on different levels of sports participation. The studies included in this review had varying enrolment criteria which is likely to have impacted the results; some of them had strict criteria (ACL-SPORTS program), while others did not. Another limitation of the included studies is a small sample size, due to one being a pilot study (Coronado et al., 2020) and other having a high drop-out rate (Joreitz et al., 2020). As previously mentioned, program adherence and skill adoption need to be considered while analyzing these studies. For example, while guided imagery is one of the techniques showing promising results (Johnson, 2000; Cupal and Brewer, 2001; Maddison et al., 2012), the RTS rates might have been higher if patients in the study by Coronado et al. (2020) had continued to practice these skills after program completion. Additionally, Coronado et al. did not asses the RTS rates beyond 6 months, so a longer follow-up would provide a clearer, less biased indication of the effectiveness of their intervention. It should be noted that the assessment of RTS rate as an outcome measure is in fact time-consuming, as these studies require a minimum follow-up of 1 year, sometimes even 2 years to allow participants to return to a preinjury level after an injury as severe as an ACL injury. However, evidence suggests that the return to any sport, return to preinjury level and especially return to competitive sport rates are different with follow-up of 2 years vs. <2 years (Ardern et al., 2011a). Another important limitation was that two of the reviewed studies did not include a control group (Coronado et al., 2020; Joreitz et al., 2020), while two studies had an active control group (Arundale et al., 2018; Capin et al., 2019). It is therefore unclear whether the RTS rate was an effect of the intervention *per se*, or the natural result of time having passed since injury.

Given that successful return to preinjury is a primary goal for all stakeholders included in elite sport (athlete, coach, parent, or even manager), further research that aims to elucidate the most effective contributing factors to a successful rehabilitation programme is warranted. Such low RTS and high reinjury rates, support a need for more comprehensive evidence-based post-rehabilitation programs, which address both the physical and psychosocial factors that determine successful RTS. To implement these strategies in the comprehensive rehabilitation protocols, there is a need for monitoring the athlete and screening for multicomponent risk factors. Moreover, to draw firm conclusions on the effects of a biopsychosocial intervention on RTS rates, future studies should be designed rigorously, with the highest methodological quality.

However, to date, research lacks evidence-based programs and studies that report RTS outcomes. Additionally, many athletes who pass RTS tests do not return to preinjury level, which is another reason to monitor RTS rates and optimize RTS tests. Research in injured athletes with dual career is also needed, as they have an additional load to balance with their education along with the trainings/rehabilitation, which could negatively affect the process of returning to sport.

## Conclusions

This review described the intervention programs used on injured athletes, while focusing on the RTS rates as an outcome. The results of exercise and psychosocial interventions were shown to be promising, with a high proportion of athletes returning to preinjury level of sport participation. Furthermore, differences were observed between males and females, with female athletes having lower RTS rates than male athletes. Although certain factors influencing RTS (e.g., age, sex, preinjury level of performance) are non-modifiable, physical and psychological function, as well as social support can be influenced by appropriate interventions. The focus of rehabilitation programmes to return to preinjury should therefore be on taking the holistic approach and addressing the aforementioned aspects.

## Data availability statement

The original contributions presented in the study are included in the article/supplementary material, further inquiries can be directed to the corresponding author/s.

## Author contributions

KD: conceptualization and writing the first draft. KD and AP: literature search and data selection. Both authors critically revised the manuscript, contributed to the article, and approved the submitted version.

## Funding

The open access publishing of this article was supported by the Slovenian Research Agency (ARRS) (project No. P5-0147 entitled The kinesiology of mono-structured, poly-structured, and conventional sports).

## Conflict of interest

The authors declare that the research was conducted in the absence of any commercial or financial relationships that could be construed as a potential conflict of interest.

## Publisher's note

All claims expressed in this article are solely those of the authors and do not necessarily represent those of their affiliated organizations, or those of the publisher, the editors and the reviewers. Any product that may be evaluated in this article, or claim that may be made by its manufacturer, is not guaranteed or endorsed by the publisher.
